# Pathology and Practical Medicine

**Published:** 1876-03

**Authors:** 


### I. Pathology and Practical Medicine.

1. *On the Formation of Pigment in Melanotic Sarcoma.* Gussenbauer. (*Virch. Arch.; Centralbl. d. Chir.*, 1876, No. 1.)

The pigment in melanotic tumors is formed at the place where it is found, but it is not derived directly from the circulating blood, nor is it the product of a specific action of the cells. This theory is sustained by these three constant symptoms: 1. The coloring matter is irregularly distributed in the melanotic growths; the pigmented portions exhibit a great variety of shades; and again, we often find an entirely colorless portion adjoining one intensely pigmented. Now the cells which constitute these tumors being all alike, and their nutritive fluid being the same, there is no plausible reason why some cells should take up or form a coloring matter while others should not. 2. The pigmented cells follow the course of the blood-vessels, and are arranged in network which closely imitates the net of the capillaries of the organ in which the melanotic tumor originated. 3. The pigmentation is always preceded by the thrombosis of blood-vessels in the circumference of the growing tumor, and the red corpuscles stored up in these thrombi, furnish the coloring matter to the neighboring cells.

According to G., the process of pigmentation takes place in this manner: Wherever pigment is found, there has first been an engorged state of the smallest blood-vessels. This condition causes a dilatation of these vessels, and also a stagnation of the blood (stasis). The stasis leads finally to a coagulation of the blood (thrombosis); but before this occurs the hæmatine has left the red cells of the stagnating blood, and, dissolved in the serum, it passes through the walls of the vessels to impregnate the neighboring parenchyma of the tumor, where it is absorbed by the cells and consolidated to a granular matter.

2. *Obliteration of the Aorta.* Lüttich. (*Arch. d. Heilkunde; Centralbl. d. Chir.*, 1876, No. 2.)

A healthy young man, aged 26, died suddenly. The post-mortem exhibited a dissecting aneurism of the ascending aorta, which, greatly dilated, showed a few atheromatous patches of its walls. The descending portion of the arch of the aorta terminated in a cul-de-sac about one inch below the origin of the left subclavian artery, and it was connected by a strong fibrous band, half an inch in length, with the blind upper end of the thoracic aorta. The obliterated ductus arteriosus passed uninterruptedly over into the fibrous band of the obliterated aorta. The innominate, the left carotid and subclavian arteries were considerably dilated. The mammary and epigastric arteries showed an increase in calibre, while the thoracic aorta was rather small.

3. *Influence of Alcohol and Acids upon the Coagulation of Blood.* ORÉ. (*Comptes rendus; Centralbl. d. Chir.*, 1876, No. 3.)

It is well known that alcohol and acids cause the blood to coagulate. But as all experiments relating to this matter have been made on blood which, though freshly drawn from the animal, at the time of the trial was outside of the organism, Oré tried to answer the question whether the *circulating* blood also is coagulated by the introduction of alcohol or acids. He experimented on dogs, and made injections into the crural vein with the following solutions: (*a.*) Vinegar and water, *aa* j. (*b.*) Sulphuric acid, 2, 5; water, 45 parts. (*c.*) Phosphoric acid, 5; water, 100 parts. (*d.*) Nitric acid, 4 parts; water, 120 parts. (*e.*) Muriatic acid, 4 parts; water, 120 parts. (*f.*) Alcohol, 7, 5 parts; water, 75 parts; and in another case, alcohol, 16; water, 75 parts. After the injections with acids, the dogs exhibited a little dyspnœa, which, however, lasted but a few minutes. When alcohol was injected, the dogs became drowsy, but showed no difficulty in respiration. A few days after the experiment, the dogs were killed, and a careful autopsy was made, in order to find the thrombi or emboli, if there were any. But in all cases the most searching examination failed to discover any coagulation of the blood. From these experiments Oré drew the conclusion, that the alcohol and the acids do not cause any coagulation if introduced into the circulating blood, and that, therefore, many substances which are not soluble in water, but can be dissolved in alcohol or in acids, could be injected into the veins without the danger of producing thrombosis or embolism.

4. *Cure of Acute Rheumatism by Salicylic Acid.* (*Boston Med. and Surg. Jour.*, February 10, 1876.)

The experiments of Prof. Traube (*Berlin Klinische Wochenschrift*) are here commented upon.

Fourteen cases of acute rheumatism were treated with the acid, and in all cases, within two days, all fever had gone, as well as the redness, swelling, and pain in the joints.

Dr. Stricker believes salicylic acid a specific for acute rheumatism, and that the results in these experiments were not simply coincidences. The acid should be the pure colorless crystals, and should be pulverized. The dose is seven to fifteen grains, and should be taken every hour until the affected joints can be moved without pain. As many as fifteen doses are usually required. The drug should be taken in wafers to protect the mouth. Increased perspiration, tinnitus aurium, deafness, and, rarely, slight mental exhilaration, are produced by the treatment.

5. *Cirrhosis of Liver in a Child.* Dr. T. D. GRIFFITHS. (*Med. Times and Gaz.*, December 18, 1875; *Mo. Abstract*, February, 1876.)

This was the case of a liver, presented to the London Pathological Society, from a female child of ten years. Dr. Griffiths was sure the child had not been addicted to drink. He had had the case under careful observation for five years. The liver weighed fifteen ounces, and was hard and tough. On its surface, granulations were fully developed. It was accepted by all the members as a case of cirrhosis, or "gin-drinker's liver."

Dr. Murchison had seen a cirrhosed liver, from a child of nine years, who had been in the habit of drinking liquor freely.

Dr. R. W. Parker had seen a boy of nine with retracted liver and ascites. He had taken grog daily at dinner.

6. *The Pathology of Pneumonia.* Dr. J. Dreschfeld. (*Lancet*, January 8, 1876; *Mo. Abstract*, February.)

From a number of experiments on rabbits, the conclusions are offered that—

1. By section of the vagi, and by injections into the lungs of nitrate of silver, an irritative inflammation is induced, similar to the acute catarrhal pneumonia in man.

2. This process consists, in the first stage, in active proliferation of the epithelial cells lining the alveoli, which become detached from the walls of the alveoli, increase in size, become granular, and, with multiplication of nuclei, produce new cells.

3. The capillaries about the alveoli become actively hyperæmic; the white corpuscles accumulate in them, and, on the detachment of the epithelium, migrate into the alveoli.

4. The epithelium, after the proliferation is completed, undergoes fatty degeneration.

This opinion is distinctive in ascribing much, in the causation of this process, to the epithelium. Dr. D. has examined many lungs with catarrhal pneumonia in children and adults, and lungs with lobular pneumonia near caseous products, and finds a close resemblance between the cells discovered and the proliferating epithelium above described.

7. *Functional Infrequency of the Pulse.* Prof. Austin Flint. (*American Practitioner*, Jan., 1876.)

Prof. Flint publishes the histories of six cases of marked infrequency of the pulse. The ages of the patients were respectively 20, 35, 32, 53, 46 and 43 years. The lowest points of infrequency reached were respectively 16, 40, 35, 26, 38 and 26 per minute. Of the five cases Flint himself had observed, the pulse in health was nearly or quite of a normal average in three; "it was presumably so in one case, and there is room for doubt in respect to this point in one case." In one case there was noted a cessation of the heart's action for sixteen and eighteen seconds. Intermittency occurred in one case, and irregularity in one.

The patients all recovered; in two of the cases, however, the pulse remained at 40 after other evidences of recovery. The author believes it *possible* for infrequency of pulse to occur as an acquired normal peculiarity, although rarely.

Of the six cases reported, in all but one the disorder of the heart was associated with "morbid cerebral disturbance." Two cases had epilepsy, with frequent epileptoid attacks. Two had mental excitability, to the extent of delirium. "In one case there was great mental and physical prostration, and gastric irritability, due apparently to cerebral disturbance." There was no organic lesion of the heart in the cases cited.

The causes of the cases were apparently as follows: Of one case, syphilitic disease; in one, the attack followed a violent fit of coughing, with spasm of the glottis; in one, the disorder occurred during convalescence from lobar pneumonia; exposure to cold and violent exercise preceded the attack in another; in one, the symptoms preceding the affection denoted malarial fever; and in the sixth case, over-ingestion and indigestion seemed to be causes.

In diagnosing this affection, Dr. F. thinks the first care should be to exclude normal infrequency, but he thinks any infrequency not normal the patient is likely to be aware of. If he is not conscious of such state when it exists, he thinks it probably normal. The second care should be to exclude organic heart lesion. Mitral lesion occasions infrequency of radial pulse, the quantity of blood by some contractions being too small to produce pulsation at the wrist. Fatty degeneration may produce the symptom under consideration. It did not exist in any of the cases detailed in the paper. Another error likely to occur is the mistaking for this a disorder characterized by a regular alternation of a strong ventricular systole with one so weak it produces no impression at the wrist. The pulse is notably infrequent in cases of intra-cranial affection, in some cases of jaundice and uræmia, and in the administration of well-known drugs.

It is believed the pathology of these cases depends on some derangement in the action of the pneumogastrics—probably an "abnormal increase in the restraining or inhibitory influence."

In the cases given, it is doubtful that the remedies employed were in any way specially efficacious. Alcohol had little or no effect in two cases treated with it; one treated awhile with bromide was not benefited, but taking strychnia, atropia and iron, and subsequently phosphorous, progressive improvement followed.

8. *Rupture of the Heart.* Dr. R. E. Van Giesen. (*N. Y. Med. Jour.*, Feb., 1876.)

A man 65 years old had never been sick in his life. While suffering severe grief at the loss of a friend he was seized with sudden vertigo. Two days afterwards Dr. V. G. saw him in an attack of nausea and vomiting, with pain in the chest. During the evening of this day he was comfortable; at one o'clock in the morning he undertook to get out of bed with a spring, when he fell dead. It was found, *post mortem*, that all the viscera except the liver, heart and aorta were healthy. The liver was large and fatty; the right ventricle was thin, and at a point near the septum there was a rupture an inch long. The heart was fatty.

Dr. V. G. had witnessed the death of a gunner, who dropped dead with heart rupture while reaching his hand for a chew of tobacco. The wall of the ventricle was as thin as tissue paper.

9. *Endemic of Pythogenic or Miasmatic-Infectious Pneumonia.* W. B. RODMAN, M.D. (*Am. Journal Med. Sc.*, January, 1876.)

There occurred in the Kentucky State Prison in 1874, 75 cases of ordinary pneumonia. The mortality was 8 per cent. From the latter part of February to July 1st, 1875, there occurred, beside several cases of ordinary pneumonia, 98 cases of a severe grade of disease which the writer calls, after Liebermeister, miasmatic-infectious pneumonia; of these cases 25 died.

This form of disease differs essentially from the common pneumonia, and is believed to be caused by "foul emanations from sewers, privies or drains, and also by men being crowded together in sleeping apartments insufficient in size and imperfectly ventilated."

At the time this epidemic occurred in the prison none was existing outside.

The cell-house of the prison seems to be miserably arranged as regards hygiene. There are six tiers of cells one above the other on each side of the house. The ventilation is bad; each cell contains only 170⅝ cubic feet of air; of course the cells are closed by grated doors. There were about 700 prisoners, and many of the cells had to be occupied each by two men. "Every man goes to his cell with a night-bucket, intended to be used only in a case of emergency; but rather than wait their turn at the privy, 400 of these men will use their buckets in their cells at various times from dark until daylight." "The lowermost tier is comparatively free from any unpleasant odor, and here there is less sickness than in any other part of the cell-house." At the top, the stench is almost unbearable. Five-sixths of the cases of this infectious pneumonia came from the top tier of cells. Dr. R. believes they were caused by the bad air of the cell-house. He does not believe the disorder is local, but general with local manifestation. Men frequently died as by malignant poison when *post mortem* examination "revealed probably only one lobe hepatized."

When the pneumonia was very slight there was sometimes violent delirium. There was often slight icterus as shown by the eye. "The pneumonia itself, both as to physical signs and *post-mortem* appearances, closely resembled an ordinary pneumonia." "In many cases almost pure blood was expectorated in large quantities, or the sputa were of a dirty brownish-black." Few patients having these symptoms recovered. The urine was brownish-red and the liver was found, *post-mortem*, to be enlarged, congested, filled with dark, syrupy blood. The average temperature seldom ranged as high as in ordinary pneumonia, nor was the pulse very much accelerated. The disease was very treacherous, and after patients seemed convalescing they would be stricken down.

The cases were first treated with carbonate of ammonia. The mortality was frightful. The tr. ferri chl. and quinia were then used, and the death-rate rapidly grew less.

Dr. R. believes that this form of pneumonia arises from miasmatic influences. It is infectious but not contagious. While a zymotic disease, it resembles others of this order, as cholera and typhoid, only in being infectious. It has different clinical features from ordinary pneumonia. (The invasion is more sudden, and it is less apt to attack the lower portion of the lung.) The disease is not so easily arrested nor so amenable to treatment as the ordinary pneumonia; many of the cases make a slow and protracted recovery.

10. *Hysteria Simulating Progressive Locomotor Ataxia.* W. H. WEBB, M.D. (*Am. Jour. Med. Sci.*, Jan., 1876.)

A woman of 35 years, who had been married at 14½, and had borne eight children beside having several miscarriages, and who had always been healthy, became melancholic apparently from a domestic mental shock in the summer of 1873. This continued until Oct. 1, following, when she had sharp shooting pains all over the body, mostly in the back and limbs, with a sense of fullness in the throat and constant desire to vomit; she had also a feeling of constriction of the chest and abdomen. Her menses now ceased, her sufferings increased, and she had pain and a peculiar feeling in the womb. Pain over the whole body now became violent, and lasted two months. In June, 1874, her arms had become gradually paralyzed. The pains were now confined to the spine and lower limbs, and lasted "some months." Then her sight became affected, she saw black spots constantly and kept in a dark room. Nothing but anodynes relieved her pains and then only in large doses, often repeated. Afterward the "arms became jerking and the fingers somewhat stiffened, with loss of power in them." Paralysis of motion and sensation occurred in the lower extremities, and control of the vesical sphincter was lost for a number of days.

Oct. 17th, 1874, she was in bed where she had lain on her back for six months. Motion was wholly lost in the upper extremities. Motion in the lower limbs was perfect while in bed; when taken out of bed and supported on both sides, she would, in attempting to walk, throw her limbs in a quick and forcible manner; there was incoördination of motion; "her feet would not come down in the place she intended they should." There was loss of sensation in the limbs. She was anæmic and emaciated, but did not appear to suffer, although she claimed to. Her pulse was 90; temperature 100° F.; urine, sp. gr. 1020, containing albumen in small quantity. Her mind had been clear during her whole sickness and she had directed her household affairs constantly. The pain in the spinal column was increased on pressure, especially over the cervical and lumbar regions, but the increase was less, when her mind was diverted from her ailment. There was some swelling over her wrists and several joints of the fingers, the parts being frequently red and painful on pressure.

On the application of the "Guiffe battery" to the various muscles of the right arm no response was obtained, and but slight tingling was complained of. Applied to the left arm, only slight muscular contraction occurred, but there was more sensation. She was given tr. ferri chloridi and acid muriat.; was thoroughly rubbed; taken out of the bed and made to walk daily. Soon she was given a carriage ride daily. By Jan. 25, 1875, she had much improved, and there was a slight return of menstruation. Feb. 13, she had three convulsions and two on the following day. She now rapidly improved, and had three regular menstruations.

By Sept. 18, she was as well as she ever was, save slight contortions of the dorsal and palmar aspects of both hands and slight contraction of her fingers.

11. *Intestinal Hæmorrhage in Typhoid Fever.* DR. TH. PLAGGE. (*Memorabilien*, xx, 11.)

The author has noticed the application of ice upon the abdomen, the administration of liq. ferri, alum, catechu, kino, tannic acid, acetate of lead, etc., to be very inefficient in hæmorrhages of the kind named above, and he is, therefore, very happy in having found a powerful and efficient remedy in ergot. His patient, a laborer, aged 32, was in the second week of typhoid fever; delirious; face flushed; tongue very dry and furred; involuntary dejections. During the night of Nov. 28, he had discharged almost a pint of pure blood, his face was pale, feet cold, pulse very feeble and frequent. He was ordered to take sherry and a mixture of extract of ergot with tincture of opium in cherry water. During that day he had three more bloody stools, but then the patient rallied and rapidly recovered.

12. *Revaccination during the Recent Epidemic of Variola in Cincinnati.* W. B. DAVIS, M.D. (*Boston Med. and Surg. Jour.*, Feb. 6, 1876.)

From Nov. 1st to Dec. 8th, 1875, and while an epidemic of variola was existing, Dr. D. revaccinated one hundred and fifty-two private patients, and fifty medical students. In 66 per cent. the revaccination was successful. In 43 per cent. the success was only partial; in 24 per cent. the vaccine disease was more or less severe. "One hundred and twenty-six persons had but one cicatrix each; of these 62 per cent. took the vaccine. Of those who had good, foveated scars, one each, 56 per cent. were successful; of those who had superficial scars, one each, 85 per cent.; of those who had distinct marks of variola and varioloid, 75 per cent.; of those who had two, three, four and six cicatrices, the number is not sufficiently large to warrant an opinion." So far as they go, "they indicate that numerous cicatrices do not give more protection than one."

In those in whom the vaccine disease is mentioned as having been "more or less severe," the disorder exactly resembled a primary vaccination.

Dr. Wm. Judkins had, during the same epidemic, revaccinated 85 cases. In 37, the effort was successful; in 48, it failed. Of the successful cases, "about half took mildly, the remainder severely." Of those vaccinated with humanized virus, 50 per cent. were successful; while of those in whom the animal virus was used, 30 per cent. were successful.

Dr. L. A. Querner had revaccinated 600 inmates of the Cincinnati Workhouse with animal virus, and 67 per cent. were successful.

Dr. D. is surprised at the measure of success in these revaccinations, and concludes that—

1. Exposure to infection and epidemic influence increases the susceptibility of the system to the influence of vaccine virus.
2. Variola and varioloid are no more protective against variola than vaccination.
3. The cicatrix is not a measure of the protection afforded by the vaccination whence it resulted.
4. It is advisable to revaccinate upon every exposure to infection, unless it has been done recently with success.
5. Those who are successfully revaccinated are to some extent susceptible to the variolous influence.

